# Authorship diversity among systematic reviews in eyes and vision

**DOI:** 10.1186/s13643-020-01451-1

**Published:** 2020-08-27

**Authors:** Riaz Qureshi, Genie Han, Kolade Fapohunda, Samuel Abariga, Renee Wilson, Tianjing Li

**Affiliations:** 1 Bloomberg School of Public Health, Johns Hopkins University, Baltimore, MD USA; 2 Department of Ophthalmology, School of Medicine, University of Colorado Anschutz Medical Campus, 1675 Aurora Ct. F731, Aurora, CO 80045 USA

**Keywords:** Systematic reviews, Eyes, Vision, Diversity, Equity, Authorship, Gender, Geography

## Abstract

**Importance:**

The inclusion of authors from diverse backgrounds and with different lived experiences is critical to ensuring the questions addressed in systematic reviews (SRs), as well as the subsequent conclusions and recommendations made, are representative of the global community.

**Objective:**

To assess the gender and geographic diversity of authors among all Cochrane SRs in eyes and vision as compared with a random sample of non-Cochrane SRs of interventions in the field of eyes and vision.

**Design:**

The Cochrane Eyes and Vision US Satellite maintains a database of SRs in the field of eyes and vision. We selected all (*n* = 313) eyes and vision intervention SRs published in *The Cochrane Library* and a random sample of 313 eyes and vision intervention SRs published elsewhere for this study. We determined gender of the first and corresponding authors (“woman,” “man,” or “unknown”) using a previously developed algorithm and their location based on institution country and the World Health Organization region.

**Results:**

From the 626 reviews included in our sample, we identified 751 unique authors who comprised 887 author positions (i.e., first and/or corresponding authors). We were able to ascertain the gender of 647/751 (86%) authors: 276 women and 371 men. Among Cochrane eyes and vision SRs, the proportions of women in first and/or corresponding author positions were consistent and approximately equal to men. Among non-Cochrane eyes and vision SRs, the representation of women was markedly lower as corresponding authors than other positions. Most authors of Cochrane eyes and vision SRs were from the UK (31%) and USA (26%), whereas most authors of non-Cochrane SRs were from China (34%).

**Conclusions and relevance:**

Compared with authors of non-Cochrane SRs in eyes and vision, authors of Cochrane SRs appear to have approximately equal representation of women and men among perceived important author positions and be located in European and North American countries, possibly due to the locations of the Cochrane editorial teams. Cochrane Eyes and Vision should continue to recruit authors from around the world in locations that reflect the global burden of eye disease.

## Background

The Cochrane Collaboration is a global organization which strives for diversity and inclusion in its membership and among its contributors [1, 2]. Diversity of Cochrane Collaboration contributors in all respects—race and ethnicity, religion, country and cultural, sexual orientation, and sex and gender identities—helps the organization develop research priorities and recommendations that reflect the needs of the global medical community [1–3].

Cochrane’s 2020 strategy includes four major goals, the fourth of which is “to be a diverse, inclusive and transparent international organization that effectively harnesses the enthusiasm and skills of our contributors” [1, 2, 4]. An Equity and Diversity Task Force has been established to address underlying challenges on the path to meeting this 2020 goal [1, 2, 4]. Geographical background has been established by Cochrane as one of the main sources of inequitable treatment within the organization [1, 4]. It is unclear whether the less than necessary geographical diversity across the organization, as indicated by Cochrane leadership, derives from not publishing enough reviews from different parts of the world, or having culturally similar authors of Cochrane reviews [4]. The terms of reference for the Equity and Diversity Task Force also note that Cochrane additionally needs to do more to address other factors of diversity, including gender.

Gender disparities in representation in authorship is well established across scientific disciplines and medical fields [5–10]. Within the field of ophthalmology, women have been found to comprise between 25 to 40% of the authors depending on the position (i.e., first author, last author, sole author, all authors) [10]. This disparity may be representative of the field overall, however, as women are underrepresented among leadership positions in ophthalmology [11–13].

In recognizing these challenges and the goals of the organization, we aimed to explore the distribution of diverse representation currently in systematic reviews from the field of eyes and vision. Specifically, we examined the gender and geographic diversity of authors in all Cochrane systematic reviews of interventions in eyes and vision as compared with a sample of non-Cochrane systematic reviews of interventions in eyes and vision.

## Methods

### Sample selection

We used a database of 4451 systematic reviews in eyes and vision maintained by the Cochrane Eyes and Vision US Satellite (CEV@US) [14]. Details of the generation of this database and its use are reported elsewhere [15–18]. We selected all Cochrane reviews of intervention in the database (*n* = 313) and randomly selected 313 non-Cochrane intervention reviews from the remaining 4138 records in the database. We selected the 313 non-Cochrane reviews by assigning each record a randomly generated, unique identifier and selected the first 313 intervention reviews. If a record was not an intervention review, we evaluated subsequent records until we found an intervention review. Although the database contains reviews with different goals, such as evaluating etiology, prevalence, or diagnostic test accuracy, we selected only intervention reviews because they are similar in purpose to Cochrane intervention reviews. As this is not a systematic review, we did not complete a PRISMA flow diagram for our study selection, nor a PRISMA checklist for the manuscript.

We restricted our evaluation to first and corresponding authors of included reviews instead of all authors. This decision was made because these author positions are associated with perceived importance and leadership in research: first authors are typically those who lead the research whereas corresponding authors are most often the senior member of the research team and occupy the last author position unless otherwise indicated [8, 19]. We restricted our assessment of diversity to gender and geographic location as these can be assessed without asking authors directly about sensitive characteristics (such as race/ethnicity/cultural identities, religion, or sexual orientation), which would require approval from the ethics committee.

### Ascertainment of gender and geographic location

To ascertain the gender and geographic location of authors, we used the authors’ name and location of their affiliation provided in the author details of the publication to conduct an internet search for any profile (e.g., academic institution, hospital staff page, LinkedIn, ResearchGate, Google Scholar, Twitter, Facebook) providing the following information: picture—used to assess gender, biography—can be used to assess gender if referenced (e.g., “She received her PhD…”), and name/country of affiliated institution. If multiple institutions were listed, we used the primary institution. If none were specified as the “primary” institution, then we used the first listed institution [20].

If none of the above types of personal profiles could be found, or a profile was lacking any of the information necessary to classify the author according to the characteristics, then any other reference to those characteristics was also acceptable. For example, news briefs or articles which feature researchers will often contain a picture of them and reference their gender and/or institution. If any characteristic could not be determined, it was classified as “unknown” for that author. Table 1 contains the specific algorithm which we previously developed and used to ascertain gender [20].

**Table 1:** Algorithm to determine author gender

**We used the following methods, in sequential order, to assign a gender to each author’s name.** 1. Editorial authors who were known to the investigator(s) 2. Names typically affiliated with a single gender (e.g., Stephanie for a woman, or Stephen for a man) 3. We searched the genderize.io web database (https://genderize.io/ accessed May 14, 2018) for names unknown to investigators and without a strong gender association, using the following steps: a. We opened a web browser. b. In the search bar, we entered the following address with the name specified: https://api.genderize.io/?name=*name* (e.g., https://api.genderize.io/?name=philip). c. The browser displayed the name, associated gender, the probability of being that gender, and the count upon which it based that probability (e.g., {“name”:“Philip”,“gender”:“male”, “probability”:1, “count”:1097}). d. We recorded the associated gender if the probability was ≥ 0.90. 4. We used the internet (e.g., using an author’s affiliation from one of their included editorials in our sample) to search for an image or reference to the author’s gender. 5. We assigned a value of “unknown” to the gender of all editorial authors for whom a gender could not be classified via methods 1-4.	

The country of each author’s affiliated institution was also given a higher-order classification based on the World Health Organization’s (WHO) Regions of the World: Europe, North America, Western Pacific, South America, South-East Asia, Eastern Mediterranean, and Africa.

### Analysis

For gender, we compared the proportions of women among unique authors and among all positions analyzed (i.e., “First and also the corresponding author”, “First authors”, and “Corresponding author”) by source of review (Cochrane vs. non-Cochrane). When comparing characteristics of authors, we considered three categories of authors to avoid double counting them: those who contributed to Cochrane reviews only, non-Cochrane reviews only, and those who contributed to both Cochrane and non-Cochrane reviews. For location, we compared the proportions of authors affiliated with institutions in the WHO regions of the world and specific countries by source of review. We calculated odds ratios for the likelihood of having a woman in an author position for Cochrane as compared with non-Cochrane reviews.

We expected to be unable to ascertain the gender of some authors included in our sample. We performed sensitivity analyses by assuming a “best” or “worst” case scenario for more equal representation, wherein respectively all of the missing genders were women or all were men. In a post hoc sensitivity analysis (as suggested by a peer reviewer), we compared the gender distribution between Cochrane and non-Cochrane reviews restricted to North American and European regions as women are traditionally underrepresented in research among Asian cultures.

## Results

### Review characteristics

Of the 626 systematic reviews included in our study, 365 (58%) listed the first author as the corresponding author; therefore, we assessed 887 total author positions (365 “First and also the corresponding authors”, 261 “First authors”, and 261 “Corresponding authors”) (Table 2). Authors could appear multiple times in our sample if they published more than one review; accordingly, we identified 751 unique authors in the 887 total author positions assessed. Most authors appeared only a single time (Table 2). The proportion of reviews with the same first and corresponding author was greater for Cochrane than non-Cochrane reviews (respectively 75% and 41%), which led the Cochrane reviews to have a larger sample of “First & corresponding authors” and non-Cochrane reviews to have a larger sample of “First authors” and “Corresponding authors” (Table 2). We also found 11 authors (three women and eight men) who contributed to both Cochrane and non-Cochrane reviews.

**Table 2:** Characteristics of authors of intervention systematic reviews in eyes and vision

Characteristics of authors	Cochrane [***n*** = 301]	Non-Cochrane [***n*** = 439]	Both [***n*** = 11]^**a**^	Total [***N*** = 751]
	***n (%)***	***n (%)***	***n (%)***	***n (%)***
**Gender**				
Woman	142 (47%)	131 (30%)	3 (27%)	276 (37%)
Man	148 (49%)	215 (49%)	8 (73%)	371 (49%)
Unknown	11 (4%)	93 (21%)	0 (0%)	104 (14%)
**Method of ascertainment**				
Knowledge of the author	30 (10%)	5 (1%)	2 (18%)	37 (5%)
Strong gender association	145 (48%)	161 (37%)	3 (27%)	309 (41%)
Genderize.io	52 (17%)	73 (17%)	1 (9%)	126 (17%)
Internet search	63 (21%)	107 (24%)	5 (46%)	175 (23%)
Not found	11 (4%)	93 (21%)	0 (0%)	104 (14%)
**Number of appearances**^**b**^				
1	263 (87%)	402 (92%)	0 (0%)	665 (89%)
2	23 (8%)	32 (7%)	8 (73%)	63 (8%)
3	8 (3%)	5 (1%)	2 (18%)	15 (2%)
4	2 (1%)	0 (0%)	0 (0%)	2 (0%)
5	2 (1%)	0 (0%)	0 (0%)	2 (0%)
7	0 (0%)	0 (0%)	1 (9%)	1 (0%)
8	2 (1%)	0 (0%)	0 (0%)	2 (0%)
10	1 (0%)	0 (0%)	0 (0%)	1 (0%)
**Author’s institution location**^**c**^				
Europe	125 (42%)	116 (26%)	6 (55%)	247 (33%)
Western Pacific	59 (20%)	181 (41%)	3 (27%)	243 (32%)
North America	88 (29%)	121 (28%)	2 (18%)	211 (28%)
South America	7 (3%)	12 (3%)	0 (0%)	19 (3%)
South-East Asia	11 (3%)	7 (2%)	0 (0%)	18 (2%)
Eastern Mediterranean	8 (3%)	2 (0%)	0 (0%)	10 (1%)
Africa	3 (1%)	0 (0%)	0 (0%)	3 (0%)
**Author’s institution country**				
China	19 (6%)	151 (34%)	2 (18%)	172 (23%)
USA	78 (26%)	90 (20%)	2 (18%)	170 (23%)
UK	95 (32%)	25 (6%)	4 (36%)	124 (17%)
Australia	25 (8%)	16 (4%)	1 (9%)	42 (6%)
Canada	10 (3%)	31 (7%)	0 (0%)	41 (5%)
Italy	7 (2%)	13 (3%)	0 (0%)	20 (3%)
Netherlands	3 (1%)	16 (4%)	0 (0%)	19 (3%)
Spain	2 (1%)	14 (3%)	0 (0%)	16 (2%)
India	10 (3%)	3 (1%)	0 (0%)	13 (2%)
Germany	4 (1%)	7 (2%)	0 (0%)	11 (1%)
Brazil	5 (2%)	5 (1%)	0 (0%)	10 (1%)
France	0 (0%)	9 (2%)	0 (0%)	9 (1%)
Singapore	6 (2%)	3 (1%)	0 (0%)	9 (1%)
Switzerland	4 (1%)	4 (1%)	0 (0%)	8 (1%)
South Korea	0 (0%)	7 (2%)	0 (0%)	7 (1%)
Austria	1 (0%)	4 (1%)	1 (9%)	6 (1%)
New Zealand	5 (2%)	1 (0%)	0 (0%)	6 (1%)
Chile	0 (0%)	5 (1%)	0 (0%)	5 (1%)
Other^d^	27 (9%)	35 (8%)	1 (9%)	63 (8%)

^a^Eleven authors contributed to both Cochrane and non-Cochrane systematic reviews

^b^Number of times an author appeared, regardless of position and journal

^c^Based on the country’s classification according to the World Health Organization regions of the world

^d^“Other” includes 32 countries with less than 5 total authors. The full listing can be found in the supplementary table

### Author gender

The majority of genders was ascertained through a strong association of the name with a particular gender (309/751, 41%) or an internet search (175/751, 23%) (Table 2). We were unable to ascertain the gender of 104/751 (14%) authors: 4% (11/301) of Cochrane authors’ genders could not be determined, but 21% (93/439) of non-Cochrane authors’ genders were unknown. Overall, women comprised 276/751 (37%) unique authors and 322/887 (36%) of all author positions analyzed, as authors could appear multiple times. Cochrane reviews had a higher proportion of unique authors who were women (142/301, 47%) compared with non-Cochrane reviews (131/439, 30%) (Table 2).

Among all “First and also the corresponding authors” (*n* = 365), 158 (43%) were women; the proportions of women authors were comparable between Cochrane (106/237, 45%) and non-Cochrane (52/128, 41%) reviews (Table 3). Among all “First authors” (*n* = 261) and “Corresponding authors” (*n* = 261), 98 (37%) and 66 (25%), respectively, were women (Table 3). This apparent discrepancy is likely due to the distribution of author types between sources as Cochrane authors comprise the majority of “First and also the corresponding authors” and have a higher proportion of women, whereas non-Cochrane authors have lower representation of women but comprised the majority of “First authors” and “Corresponding authors”. For these two author positions (i.e., first or corresponding author), Cochrane reviews had a greater proportion of women—respectively 40/76 (53%) and 35/76 (46%)—than non-Cochrane reviews—respectively 58/185 (31%) and 31/185 (17%) (Table 3). The odds of having a woman in the positions of “First and also the corresponding author”, “First author”, and “Corresponding author” were 1.14 (95% CI 0.72 to 1.82), 1.50 (95% CI 0.81 to 2.77), and 3.67 (95% CI 1.90 to 7.07) for Cochrane reviews as compared to non-Cochrane reviews, respectively.

**Table 3:** Characteristics of Cochrane and non-Cochrane systematic reviews in eyes and vision

	Cochrane systematic reviews	Non-Cochrane systematic reviews	Total
	***n*** (%)	***n*** (%)	***n*** (%)
**Intervention systematic reviews**	***n*** **= 313**	***n*** **= 313**	***n*** **= 626**
**Same first and corresponding author**			
Yes	237 (75%)	128 (41%)	365 (58%)
No^a^	76 (25%)	185 (59%)	261 (42%)
**All author positions**^**b**^			
**Gender of author (first and corresponding)**^**c**^	***n*** **= 237**	***n*** **= 128**	***n*** **= 365**
Woman	106 (45%)	52 (41%)	158 (43%)
Man	125 (53%)	70 (55%)	195 (53%)
Unknown	6 (2%)	6 (5%)	12 (3%)
**Gender of author (first only)**^**d**^	***n*** **= 76**	***n*** **= 185**	***n*** **= 261**
Woman	40 (53%)	58 (31%)	98 (37%)
Man	34 (45%)	74 (40%)	108 (41%)
Unknown	2 (3%)	53 (29%)	55 (21%)
**Gender of author (corresponding only)**^**e**^	***n*** **= 76**	***n*** **= 185**	***n*** **= 261**
Woman	35 (46%)	31 (17%)	66 (25%)
Man	36 (47%)	117 (63%)	153 (59%)
Unknown	5 (7%)	37 (20%)	42 (16%)
**“Best-case” sensitivity analysis: all unknown author genders are “women”**			
**Gender of author (first and corresponding)**	***n*** **= 237**	***n*** **= 128**	***n*** **= 365**
Woman	112 (47%)	58 (45%)	170 (47%)
Man	125 (53%)	70 (55%)	195 (53%)
**Gender of author (first only)**	***n*** **= 76**	***n*** **= 185**	***n*** **= 261**
Woman	42 (55%)	111 (60%)	153 (59%)
Man	34 (45%)	74 (40%)	108 (41%)
**Gender of author (corresponding only)**	***n*** **= 76**	***n*** **= 185**	***n*** **= 261**
Woman	40 (53%)	68 (37%)	108 (41%)
Man	36 (47%)	117 (635)	153 (59%)
**“Worst-case” sensitivity analysis: all unknown author genders are “men”**			
**Gender of author (first and corresponding)**	***n*** **= 237**	***n*** **= 128**	***n*** **= 365**
Woman	106 (45%)	52 (41%)	158 (43%)
Man	131 (55%)	76 (59%)	207 (57%)
**Gender of author (first only)**	***n*** **= 76**	***n*** **= 185**	***n*** **= 261**
Woman	40 (53%)	58 (31%)	98 (38%)
Man	36 (47%)	127 (69%)	163 (62%)
**Gender of author (corresponding only)**	***n*** **= 76**	***n*** **= 185**	***n*** **= 261**
Woman	35 (46%)	31 (17%)	66 (25%)
Man	41 (54%)	154 (83%)	195 (75%)

^a^If it was unclear whether the first author was also the corresponding author, then the senior (i.e., last) author was considered the corresponding author

^b^This section includes all first and corresponding author positions (i.e., the same author may appear multiple times in any of these positions); thus, the total denominator is 887, not 751 (i.e., unique authors)

^c^Total denominator = 365 (237 for Cochrane and 128 for non-Cochrane systematic reviews: included only first authors that were also corresponding authors)

^d^Total denominator = 261 (76 for Cochrane and 185 for non-Cochrane systematic reviews: included only first authors that were not also corresponding authors)

^e^Total denominator = 261 (76 for Cochrane and 185 for non-Cochrane systematic reviews: included only corresponding authors that were not also first authors)

The gender of “First author” and “Corresponding author” positions in non-Cochrane reviews had a substantial amount of uncertainty—respectively 53/185 (29%) and 37/185 (20%) were unknown—as compared with authors of Cochrane reviews for which only 2/76 (3%) and 5/76 (7%) of authors’ genders were unknown (Table 3).

In our sensitivity analyses, in which we assumed the extreme situations of women comprising all or none of the missing genders, we found that representation of women among Cochrane authors did not substantially change (Table 3). Among non-Cochrane authors, under a best-case assumption that all missing author genders were women, the representation of women met or surpassed among Cochrane authors for two of the categories (Table 3). Among corresponding authors for non-Cochrane reviews, however, under the extreme assumption of all missing author genders being women, only 68/185 (37%) would be women (Table 3). The odds ratios revealed similar patterns under these sensitivity analyses with only the corresponding author position being held by women significantly more often for Cochrane reviews than non-Cochrane reviews under both best-case (OR = 1.91, 95% CI 1.07 to 3.40) and worst-case (OR = 4.24, 95% CI 2.24 to 8.00) assumptions.

### Author location

**Fig. 1:**
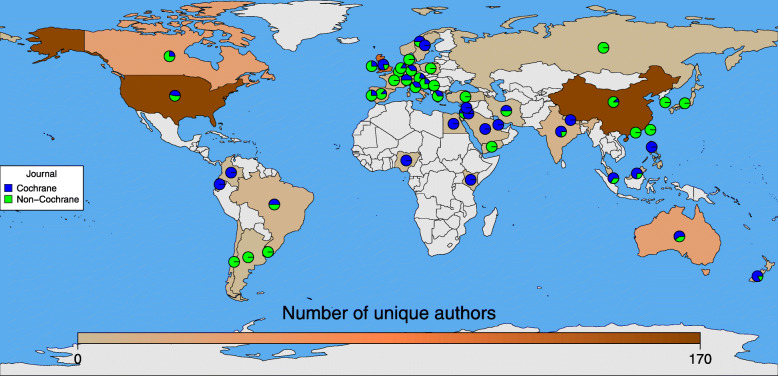
Global locations and numbers of first and corresponding authors of Cochrane and non-Cochrane systematic reviews from 50 countries. Note: The light gray color indicates no data (i.e., no authors with affiliations in that country). Maximum number of unique authors is 170 because we did not include authors contributing to both Cochrane and non-Cochrane reviews in the generation of the figure so as to not double count them (e.g., two authors from China contributed to both and thus the maximum is reduced from 172 to 170). See Table 2 for exact numbers and proportions of authors in each country, including the 11 authors who contributed to both Cochrane and non-Cochrane reviews

We were able to identify the geographic location for all authors based on their institutional affiliation. Most authors for the reviews included in our sample had institutions in either European (247/751; 33%), Western Pacific (243/751; 32%), or North American (211/751; 28%) regions of the world. As compared to non-Cochrane reviews, Cochrane reviews had a greater proportion of authors from Europe—respectively 125/301 (41%) and 116/439 (26%)—and a lower proportion from the Western Pacific region—respectively 59/301 (20%) and 181/439 (41%) (Table 2 and Fig. 1). Other parts of the world were uncommon contributors of authors to reviews in eyes and vision, with Africa being the least frequently appearing region with only three authors hailing from African countries (Table 2 and Fig. 1).

The specific countries which contributed the most authors to reviews in our sample were China (172/751, 23%), the USA (170/751, 23%), and the UK (124/751, 17%). Between these three countries, Cochrane reviews had more authors from the USA (78/301, 26%) and UK (95/301, 31%) than China (19/301, 6%), whereas non-Cochrane reviews had the opposite with 151/439 (34%) of authors being located in China and 90/439 (20%) and 25/439 (6%) respectively located in the USA and the UK.

To explore the possibility that the differences in gender distribution observed between Cochrane and non-Cochrane reviews were influenced by the location of contributing authors, we examined the gender among the 458 unique authors from Europe and North America. We found the gender representation was equal among the Cochrane authors (49% women, 50% men, 1% unknown), but there was a disparity in the representation of women among non-Cochrane authors (39% women, 60% men, 1% unknown).

## Discussion

We found differences between Cochrane and non-Cochrane reviews in the field of eye and vision research with regard to the diversity of authors in lead position (i.e., first and/or corresponding) in terms of geographic location and gender representation. It should be noted that as Cochrane reviews are managed by Cochrane review groups, which can have their own defined rules for authorship (including who may be the corresponding author), some differences we observed between Cochrane and non-Cochrane reviews may be expected. Indeed, a systematic difference between these two sources of reviews which is relevant to our assessment is that Cochrane reviews tended to have more first authors who were also corresponding authors than did non-Cochrane reviews, which meant that the actual sample of unique authors was smaller for Cochrane reviews than non-Cochrane reviews (respectively 301 and 439 unique authors).

With regard to gender, we found that both Cochrane and non-Cochrane reviews in the field of eye and vision research had approximately equal representation of gender among first authors who were also the corresponding authors, but that non-Cochrane reviews were less likely to have women than men among authors who had a single role as either first or corresponding author. Our sensitivity analyses revealed similar possible gender representation for Cochrane reviews under either scenario but widely different possible representations for non-Cochrane reviews. This suggests that Cochrane eyes and vision reviews have a good representation of women in leading author positions that is better than non-Cochrane reviews among corresponding authors and comparable to non-Cochrane reviews for other positions.

That Cochrane has truly established a sense of gender equity by having good representation of women in leading author positions, however, as opposed to simply reflecting the current gender distribution in the field of eyes and vision or a difference between Cochrane and non-Cochrane reviews influenced by the location of contributing authors, requires support. We found supporting evidence in our examination of the gender distribution of Cochrane and non-Cochrane authors from Europe and North America where a disparity in representation of women remained between Cochrane and non-Cochrane reviews. This suggests that even in cultures where women are not traditionally underrepresented in research, Cochrane has a more diverse authorship. Further support for Cochrane’s equity is found in the gender distribution among leadership positions within ophthalmology which are predominantly held by men, including among academics from whom we would expect literature contributions in important author roles [11–13].

There is a substantial amount of literature to support that women are underrepresented in leadership positions in academia [5, 6, 8, 19–24]. This is independent of field and has been documented even in fields which are predominantly comprised of women, such as pediatrics [7]. With regard to ophthalmology specifically, our findings are very similar to a recent study of multiple medical fields which found 36% of first author (compared with our 37% of all, Cochrane and non-Cochrane, first authors), and 24% of last author positions (compared with our 25% of all corresponding author positions which were most often last authors) to be held by women [10]. Trends in authorship have been used in the past as a proxy for leadership in a field, particularly authorship of editorials and opinion pieces [6, 8, 20–22, 25]. Many investigations also use authorship in field-specific journals as a proxy for a specific field. As compared with many other areas of medicine, including ophthalmology, our study shows that Cochrane eyes and vision reviews have a good representation of women in important leadership positions. The underrepresentation of women in corresponding author positions among non-Cochrane reviews could be due to having few women in senior academic positions in the field of eyes and vision [11–13]. This is an issue which would require more in-depth assessment of the field as a whole and is beyond the scope of this work.

Geographically, Cochrane authors largely came from North America or European countries whereas non-Cochrane authors were highly concentrated in the Western Pacific region of the world. Each of these three regions had one country which contributed the most to the author pool and those were respectively the USA, the UK, and China. It is likely that the Cochrane authors are primarily found in these countries due to the locations of the editorial teams: in London, UK; Baltimore, Maryland, USA (recently moved to Denver, Colorado, USA); and Florence, Italy. However, the number of authors for non-Cochrane reviews located in China was surprising. Only 6% of Cochrane authors had their primary affiliation in China whereas 34% of the non-Cochrane authors were located in China. This suggests that perhaps Cochrane Eyes and Vision would do well to set their sights on China and other Western Pacific nations as a pool for potential authors and perhaps aim to establish a center in the region or recruit more authors from the area. Further, although Europe was the predominant source of Cochrane authors, they were mostly located in the UK and several European countries had higher proportions of non-Cochrane authors which may suggest that these countries also have potentially untapped potential with regard to contributions to Cochrane. This finding is echoed in a recent investigation of Cochrane Eyes and Vision Cataract reviews which found only 17% of Cochrane reviews assessed included an author from a low- and middle-income country in their byline [26].

Cochrane reviews have been consistently found to be of higher methodological quality than non-Cochrane reviews [14, 27–29]. Recruiting and training these authors in Cochrane methods would greatly increase the capacity of Cochrane Eyes and Vision research output and the geographic diversity and representation in Cochrane reviews, as well as improve the overall quality of the evidence for eyes and vision conditions [15–18].

This research is not without limitations, primarily in the method of ascertaining gender and the restriction to first and corresponding author positions. We used an algorithm which we had previously developed and tested to ascertain the gender of included authors [20]. Although the algorithm has been shown to be highly sensitive and specific to a gender binary, we did not categorize gender on a spectrum [20]. There is always a potential for misclassification of gender; however, any misclassification is likely to be non-differential in nature and unlikely to be large or change conclusions because the sensitivity and specificity are equal for men and women and the prevalence of non-binary genders is low. A related limitation was the substantial amount of missingness in the genders of our non-Cochrane authors which could have affected comparisons between the sources of reviews; however, we conducted a sensitivity analyses and discussed the associated results.

## Conclusions

Cochrane reviews in eyes and vision appear to have equal representation of women and men among the first and corresponding authors. Compared with non-Cochrane reviews in eyes and vision, Cochrane reviews appear to have a greater concentration of authors who are based in European and North American countries. The number of systematic reviews in eyes and vision being produced in China reflects a large burden of eye disease in Asia. Cochrane Eyes and Vision should continue to recruit authors from around the world in locations that reflect the global burden of eye disease.

## Supplementary information

**Additional file 1: Supplementary Table.** Countries of 751 unique authors of intervention systematic reviews in eyes and vision

## Data Availability

The datasets used and/or analyzed during the current study are available from the corresponding author on reasonable request.

## Abbreviations

CEV@US: Cochrane Eyes and Vision United States Satellite
WHO: World Health Organization
USA: United States of America
UK: United Kingdom
