# Supplementary material for: Authorship diversity among systematic reviews in eyes and vision
Source: Syst Rev. 2020 Aug 27;9:192. doi: 10.1186/s13643-020-01451-1 (PMC7450569; doi:10.1186/s13643-020-01451-1)
Supplement: Supplementary file 1 — Additional file 1: Supplementary Table. Countries of 751 unique authors of intervention systematic reviews in eyes and vision [file 13643_2020_1451_MOESM1_ESM.docx]

**Supplementary Table.** Countries of 751 unique authors of intervention systematic reviews in eyes and vision

| **Characteristics of**  **unique authors** | **Cochrane**  **[n = 301]** | | **Non-Cochrane**  **[n = 439]** | | **Both ^a^**  **[n = 11]** | | **Total**  **[N = 751]** | |
| --- | --- | --- | --- | --- | --- | --- | --- | --- |
|  | **n** | **(%)** | **n** | **(%)** | **n** | **(%)** | **n** | **(%)** |
| **Author’s institution country** |  |  |  |  |  |  |  |  |
| China | 19 | (6%) | 151 | (34%) | 2 | (18%) | 172 | (23%) |
| United States of America | 78 | (26%) | 90 | (20%) | 2 | (18%) | 170 | (23%) |
| United Kingdom | 95 | (32%) | 25 | (6%) | 4 | (36%) | 124 | (17%) |
| Australia | 25 | (8%) | 16 | (4%) | 1 | (9%) | 42 | (6%) |
| Canada | 10 | (3%) | 31 | (7%) | 0 | (0%) | 41 | (5%) |
| Italy | 7 | (2%) | 13 | (3%) | 0 | (0%) | 20 | (3%) |
| Netherlands | 3 | (1%) | 16 | (4%) | 0 | (0%) | 19 | (3%) |
| Spain | 2 | (1%) | 14 | (3%) | 0 | (0%) | 16 | (2%) |
| India | 10 | (3%) | 3 | (1%) | 0 | (0%) | 13 | (2%) |
| Germany | 4 | (1%) | 7 | (2%) | 0 | (0%) | 11 | (1%) |
| Brazil | 5 | (2%) | 5 | (1%) | 0 | (0%) | 10 | (1%) |
| France | 0 | (0%) | 9 | (2%) | 0 | (0%) | 9 | (1%) |
| Singapore | 6 | (2%) | 3 | (1%) | 0 | (0%) | 9 | (1%) |
| Switzerland | 4 | (1%) | 4 | (1%) | 0 | (0%) | 8 | (1%) |
| South Korea | 0 | (0%) | 7 | (2%) | 0 | (0%) | 7 | (1%) |
| Austria | 1 | (0%) | 4 | (1%) | 1 | (9%) | 6 | (1%) |
| New Zealand | 5 | (2%) | 1 | (0%) | 0 | (0%) | 6 | (1%) |
| Chile | 0 | (0%) | 5 | (1%) | 0 | (0%) | 5 | (1%) |
| Croatia | 1 | (0%) | 3 | (1%) | 0 | (0%) | 4 | (1%) |
| Denmark | 0 | (0%) | 3 | (1%) | 1 | (9%) | 4 | (1%) |
| Ireland | 1 | (0%) | 3 | (1%) | 0 | (0%) | 4 | (1%) |
| Israel | 2 | (1%) | 2 | (0%) | 0 | (0%) | 4 | (1%) |
| Malaysia | 3 | (1%) | 1 | (0%) | 0 | (0%) | 4 | (1%) |
| Portugal | 1 | (0%) | 3 | (1%) | 0 | (0%) | 4 | (1%) |
| Greece | 1 | (0%) | 2 | (0%) | 0 | (0%) | 3 | (0%) |
| Hong Kong | 0 | (0%) | 3 | (1%) | 0 | (0%) | 3 | (0%) |
| Saudi Arabia | 3 | (1%) | 0 | (0%) | 0 | (0%) | 3 | (0%) |
| Iran | 1 | (0%) | 1 | (0%) | 0 | (0%) | 2 | (0%) |
| Nigeria | 2 | (1%) | 0 | (0%) | 0 | (0%) | 2 | (0%) |
| Norway | 1 | (0%) | 1 | (0%) | 0 | (0%) | 2 | (0%) |
| Poland | 0 | (0%) | 2 | (0%) | 0 | (0%) | 2 | (0%) |
| Sweden | 2 | (1%) | 0 | (0%) | 0 | (0%) | 2 | (0%) |
| Taiwan | 0 | (0%) | 2 | (0%) | 0 | (0%) | 2 | (0%) |
| Turkey | 0 | (0%) | 2 | (0%) | 0 | (0%) | 2 | (0%) |
| Argentina | 0 | (0%) | 1 | (0%) | 0 | (0%) | 1 | (0%) |
| Bahrain | 1 | (0%) | 0 | (0%) | 0 | (0%) | 1 | (0%) |
| Belgium | 0 | (0%) | 1 | (0%) | 0 | (0%) | 1 | (0%) |
| Colombia | 1 | (0%) | 0 | (0%) | 0 | (0%) | 1 | (0%) |
| Ecuador | 1 | (0%) | 0 | (0%) | 0 | (0%) | 1 | (0%) |
| Egypt | 1 | (0%) | 0 | (0%) | 0 | (0%) | 1 | (0%) |
| Japan | 0 | (0%) | 1 | (0%) | 0 | (0%) | 1 | (0%) |
| Jordan | 1 | (0%) | 0 | (0%) | 0 | (0%) | 1 | (0%) |
| Kenya | 1 | (0%) | 0 | (0%) | 0 | (0%) | 1 | (0%) |
| Lebanon | 1 | (0%) | 0 | (0%) | 0 | (0%) | 1 | (0%) |
| Nepal | 1 | (0%) | 0 | (0%) | 0 | (0%) | 1 | (0%) |
| Philippines | 1 | (0%) | 0 | (0%) | 0 | (0%) | 1 | (0%) |
| Russia | 0 | (0%) | 1 | (0%) | 0 | (0%) | 1 | (0%) |
| Serbia | 0 | (0%) | 1 | (0%) | 0 | (0%) | 1 | (0%) |
| Uruguay | 0 | (0%) | 1 | (0%) | 0 | (0%) | 1 | (0%) |
| Yemen | 0 | (0%) | 1 | (0%) | 0 | (0%) | 1 | (0%) |

^a^ Contributed to both Cochrane and Non-Cochrane SRs
